# Insights on the Impact of External and Internal Boosting on Varicella-Zoster Virus Reactivation Based on Evidence From the First Decade of the United States Universal Varicella Vaccination Program

**DOI:** 10.7759/cureus.16963

**Published:** 2021-08-06

**Authors:** Gary S Goldman

**Affiliations:** 1 Research, Independent Computer Scientist, Bogue Chitto, USA

**Keywords:** exogenous exposures, herpes zoster, herpes zoster incidence, universal varicella vaccination, varicella, varicella vaccine efficacy, capture-recapture, varicella-zoster virus

## Abstract

Since the licensure of the varicella vaccine in the United States in 1995 and the implementation of the universal varicella vaccination program, varicella infection rates, and associated morbidity and mortality rates have decreased. However, controversy exists over whether universal vaccination has resulted in an increased incidence of herpes zoster (HZ). In 1965, Dr. Hope-Simpson hypothesized that exogenous exposures to the wild-type varicella-zoster virus (wt-VZV) provide immune boosts that inhibit HZ; therefore, reducing the amount of circulating wt-VZV may have the negative effect of increasing the incidence of HZ. A historical review of data from the Centers for Disease Control and Prevention-sponsored Antelope Valley Varicella Active Surveillance Project, along with other studies, is provided to investigate the exogenous boosting hypothesis in the first decade post-vaccine licensure. These data indicated that adoption of universal varicella vaccination led to (1) significant HZ incidence rate increases among children, adolescents, and adults with a history of wild-type varicella and (2) decline in varicella vaccine efficacy after the initial post-licensure period. These effects were likely due to reduced exogenous exposures from children shedding wt-VZV. Appropriate methodologies for ongoing research are also discussed, both in studies during the first decade post-licensure and more recent work.

## Introduction and background

Varicella-zoster virus and initiation of the universal vaccination program

The varicella-zoster virus (VZV) is responsible for both varicella (chickenpox) and herpes zoster (HZ) (shingles). Primary infection with VZV as varicella usually occurs during childhood and generally causes mild disease. Serious complications are more common when primary infection occurs in infancy (≤18 months) or adulthood. After symptoms resolve, VZV remains in latent form in sensory ganglia and may reactivate to cause HZ, especially as immunity decreases over the individual’s lifetime or if the individual is immunocompromised.

In 1965, Dr. Hope-Simpson, a British general practitioner who meticulously tracked cases of HZ occurring in his clinical practice in Cirencester for 16 years, reported age-stratified HZ incidence rates. The step-wise increases in HZ incidence rates in successively older age categories likely led to his hypothesis that exposure to circulating VZV and incidence of HZ were inversely linked. He suggested that two mechanisms--exogenous exposures (i.e., outside boosting) and asymptomatic endogenous reactivations (i.e., internal boosting)--were involved in boosting immunity [1]. Herein lies the controversy: how significant were exogenous exposures in boosting cell-mediated immunity to inhibit the reactivation of VZV as HZ? If, on the other hand, internal boosting were significant, then the decline in exogenous exposures concomitant with universal varicella vaccination would have minimal effect on HZ incidence rates. Although the controversy remains, since Dr. Hope-Simpson’s original hypothesis, several studies have provided evidence that repeated exogenous exposures play the predominant role in boosting immunity and preventing or postponing HZ, compared to subclinical internal boosting (i.e., endogenous reactivation) of latent VZV [2-8].

Despite prior concerns that reducing exogenous exposures to wild-type varicella might increase the incidence of HZ, the United States licensed the varicella vaccine and recommended it for universal use in 1995. The Summary for Basis of Approval Agreement between the Food and Drug Administration (FDA) and Merck (the varicella vaccine manufacturer) acknowledged the concern "that universal vaccination might result in increased rates of herpes zoster in vaccinated and unvaccinated individuals" [9]. A 1995 special report published in the *Journal of Pediatrics* also laid out the concerns about the effect of universal varicella vaccination on HZ incidence:

The incidence of zoster in vaccinated and unvaccinated individuals might increase after universal immunization. There is evidence that reexposure to natural chickenpox boosts cellular immunity and potentially reduces an individual's likelihood of having zoster. Vaccine-induced herd immunity will reduce exposure to wild-type varicella; mathematical modeling indicates that the frequency of zoster in adults could increase [10].

Despite concerns acknowledged by the FDA, Merck, and other health authorities regarding the effect that a loss of exogenous boosts following universal varicella vaccination might have on the incidence of HZ, universal varicella vaccination was adopted in the United States. The program was expected to be cost-effective, with cost savings from decreasing parental time lost from work to care for a child with chickenpox [11]. The initial cost-benefit analysis was based on the assumptions that one vaccine at a cost of $35 would provide lifetime protection against varicella and that universal varicella vaccination would have a negligible impact on the closely related HZ epidemiology. To address the concerns about a potential rise in HZ cases while still decreasing the health burden of varicella, an alternative to universal vaccination would be to administer the varicella vaccine to children aged 12 years who remain susceptible to varicella. Implementing this strategy would prevent varicella in adults, where the disease is often severe, and preserve the subclinical boosts to cell-mediated immunity gained from periodic exogenous exposures to the circulating wild-type VZV (wt-VZV), thus averting increases in adult HZ incidence (beyond baseline trends) and the associated medical costs required for treatment.

## Review

Impact of universal varicella vaccination in the first decade post-licensure: The Antelope Valley Varicella Active Surveillance Project

In late 1994, the Centers for Disease Control and Prevention (CDC) and the Los Angeles County Department of Health Services (LADHS) formed a cooperative agreement to establish the Antelope Valley (AV) Varicella Active Surveillance Project (VASP). This project’s mission was to perform epidemiological studies and monitor the effects of the universal varicella vaccination program on the 300,000 residents comprising the study population within the AV region (principally two cities, Lancaster and Palmdale in California), beginning in 1995. Dr. Goldman, Research Analyst for VASP, in response to long-time nurses reporting for the first time in 1999 that they were encountering a surprising number of HZ cases among school-aged children, proposed adding active surveillance of HZ. With CDC approval, HZ surveillance began in January 2000. Goldman resigned in October 2002 due to concerns that the study data were not being presented accurately.

Baseline HZ data

Ideally, the same population-based, active surveillance data should be used to compute and compare HZ incidence rates both pre- and post-varicella vaccine licensure. Unfortunately, CDC did not initially authorize VASP to conduct active surveillance of HZ. Thus, no baseline HZ incidence data exists for the AV study region during the pre-licensure (pre-1995) and early post-licensure years (1995-1999). However, there are three "surrogate" HZ incidence rates available--two from historical studies in other populations [1,12], and one from a VASP-sponsored survey conducted among parents of middle-school students aged 10 to 14 years within the AV population [13]. HZ incidence rates in the VASP-sponsored study were remarkably consistent with those reported in the Hope-Simpson and Donahue studies (Table 1).

**Table 1:** HZ incidence rates (cases/100,000 p-y) among individuals with a history of wild-type varicella aged <20 years in the pre- and early post-varicella vaccine licensure periods CI: confidence interval; p-y: person-years; VASP: Varicella Active Surveillance Project ^a^Hope-Simpson computed crude HZ incidence rates that included observation time among individuals who had never had varicella. However, since few children in the 10-19-year age-category remain susceptible to varicella, the true and crude incidence rates are similar.

Study	Cumulative years	Age (years)	Observation time (p-y)	HZ incidence rate (95% CI)
VASP-sponsored survey[13]	1986-1995	<10	12,457	144 (86-228)
	1996-2000	10-14	16,792	125 (78-191)
	1986-2000 combined	<15	29,249	133 (95-182)
Hope-Simpson^a^ [1]	1947-1962	10-19	7,280	138 (74-255)
Donahue *et al.* [12]	1990-1992	<14	36,842	133 (98-176)

The three methodologically different studies, conducted in dissimilar populations, during different pre-varicella vaccine time periods, reported nearly identical HZ incidence rates among individuals aged <20 years. Despite limitations inherent to the VASP-sponsored survey--which investigated susceptibility to varicella and HZ incidence rates--respondents reflected the socio-economic and racial balance of the AV population and the collected data appear to have accurately captured the true HZ incidence rates among children during the pre-varicella vaccine licensure years and among individuals aged 10-14 years in the early post-licensure period [13].

Increased HZ incidence rates after vaccine licensure among children, adolescents, and adults with a history of varicella

By 2000, the varicella vaccine had been administered to approximately 50% of children aged <10 years in the Antelope Valley study population. Varicella incidence had declined by 80% and the characteristic seasonal peaks were no longer distinguishable. Thus, the VASP was positioned to acquire HZ case reports among children, adolescents, and adults who were now predominantly experiencing internal boosts (asymptomatic endogenous reactivations) in the near absence of exogenous boosts. These post-licensure conditions allowed Dr. Hope-Simpson’s 1965 hypothesis to be tested.

Using Hope-Simpson as a reference, HZ incidence rates in the pre-varicella vaccine licensure era were more accurately represented by a relatively constant true HZ incidence rate (using rounded figures) of 140 cases per 100,000 p-y among individuals aged 1-19, then nearly doubling to 260 cases per 100,000 p-y among adults aged 20-49, then nearly doubling again to 500 cases per 100,000 p-y among adults aged 50-59 [1]. Noting that pre-licensure HZ incidence rates strictly increased in age-stratified groups ranging from youngest to oldest, puts into better perspective Hope-Simpson’s 1965 hypothesis [1]: "The peculiar age distribution of zoster may in part reflect the frequency with which the different age groups encounter cases of varicella, and because of the ensuing boost to their antibody protection have their attacks of zoster postponed." According to the exogenous boosting hypothesis, aging adults experience progressively fewer exogenous exposures to children shedding VZV, resulting in higher HZ incidence rates. This normally decreasing frequency of exogenous exposures with age was accelerated and maximized following implementation of the universal varicella vaccination program as vaccination rates increased and the growing proportion of varicella vaccinees diminished the remaining population of children shedding wt-VZV.

From 2000 to 2001, HZ cases reported to VASP either maintained or increased in every adult 10-year age category (Table 2), with a statistically significant increase in HZ case reports of 28.5%, from 158 in 2000 to 203 in 2001 (paired t-test: p<0.042, t=2.95, df=4) [14,15].

**Table 2:** Adult HZ case reports stratified by 10-year age categories, VASP, 2000–2001 HZ: herpes zoster; VASP: Varicella Active Surveillance Project ^a^The paired t-test indicates a statistically significant increase in HZ case reports from 2000 to 2001 (p<0.042; t=2.95; df=4).

	Adult age category (years)					
Year of surveillance	20-29	30-39	40-49	50-59	60-69	Total
2000	10	20	50	43	35	158
2001	19	27	50	62	45	203
Percent change	90.0%	35.0%	0.0%	44.2%	28.6%	28.5%^a^

The 2000-2001 increase in reported HZ cases among adults was a preliminary indication that exogenous exposures might play a significant role in maintaining immunity. In subsequent years, the VASP Co-Principal Investigators periodically added additional surveillance units, reasoning that a more exhaustive collection of adult HZ cases would enhance data analysis. On the contrary, the absence of a "control" of a consistent base of reporting sites prevented further analysis of relative trends in annual adult HZ incidence rates. On this issue, epidemiological statistics expert Dr. Tilling clarifies:

We also need to move away from the idea that all registers need to be as complete as possible. A well-designed, incomplete, register may provide a more accurate, unbiased estimate of incidence than a nearly complete register which fails to identify particular population groups [16].

After another CDC-sponsored study [17] reported a statistically significant (p<0.001) increase in HZ incidence of 90% (or 22.5% annually), from 277 cases/100,000 p-y in 1999 to 525 cases/100,000 p-y in 2003, CDC sponsored no other *prospective* studies of adult or population HZ incidence rates *in communities with widespread varicella vaccination* for nearly two decades. 

In addition to increasing HZ incidence rates among adults, rates among children with a history of varicella were unusually high. The ascertainment-corrected HZ incidence rate among children aged <10 years with a history of wild-type varicella was 484 cases/100,000 p-y for cumulative years 2000-2001 (Table 3)--more than three-fold higher than the pre-licensure rates derived from the VASP-sponsored survey [13] and reported in the study by Donahue et al. [12]

**Table 3:** HZ incidence rates (cases/100,000 person-years) among children with a history of wild-type varicella during 2000, 2001, and cumulative (2000-2001), VASP Abbreviations: CI — confidence interval; HZ – herpes zoster; p-y – person-years

Year	Age (years)	Observation time (p-y)	HZ incidence rate (95% CI)	Ascertainment-corrected HZ incidence rate
2000	<10	16,127	236 (167-323)	471
2001	<10	10,751	251 (169-371)	502
Cumulative (2000-2001)	<10	26,878	242 (186-308)	484

Interestingly, the cumulative 2000-2003 ascertainment-corrected HZ incidence rate of 122 cases/100,000 p-y among adolescents aged 10 to 19 years (Table 4) reported by Goldman [18] was approximately the same as the cumulative pre-licensure and early post-licensure (1986-2000) rates derived from the VASP-sponsored survey (Table 1). Presumably, adolescents were recently boosted via exogenous exposures and cell-mediated immunity had not declined to levels below a protective threshold. However, among adolescents, VASP reported a 65% HZ incidence rate increase from 2000 to 2006, which was still increasing through 2012 [19,20].

**Table 4:** Comparison of cumulative HZ incidence rates (cases/100,000 person-years) among children and adolescents reported by CDC/VASP and Goldman Abbreviations: CI – confidence interval; HZ – herpes zoster; p-y — person-years; VASP – Varicella Active Surveillance Project ^a^CDC/VASP authors’ 2000-2006 unadjusted HZ incidence rates (based on raw counts of HZ case reports) confirm Goldman's 2000-2003 findings. ^b^Capture-recapture estimated 50% reporting completeness. ^c^Based on 21 cases reported during an observation time of 152,250 p-y. ^d^Based on 94 cases reported during an observation time of 42,096 p-y.

Varicella exposure history (Age in years)	CDC-reported [20]	Goldman-reported [18]	
	Cumulative 2000-2006 HZ incidence rate unadjusted (95% CI)	Cumulative 2000-2003 HZ incidence rate unadjusted (95% CI)^a^	Ascertainment-corrected^b^ HZ incidence rate
Vaccinated children, 1-9	19 (15 - 25)	13.8^c^ (9 - 21)	27.6
Children with natural disease, 1-9	239 (193 - 295)	223^d^ (180 - 273)	446
Adolescents with natural disease, 10-19	69 (61 - 77)	61 (51 - 72)	122

The cumulative 2000-2003 ascertainment-corrected HZ incidence rate of 27.6 cases/100,000 p-y among vaccinated children aged 1-9 years reported by Goldman [18] (Table 4) was lower than reported pre-licensure rates (among unvaccinated children) and closely agreed with another US study that reported a cumulative 2007-2008 rate of 27.4 cases/100,000 p-y (95% C.I. 22.7-32.7) among vaccinated children aged ≤12 years [21]. This notably low HZ incidence rate among vaccinees served as a control that cases of HZ were not being over-diagnosed. Vaccinees reflected a low HZ incidence rate because of the lower risk of reactivation of the vaccine strain of VZV (v-VZV) compared with reactivation of wt-VZV. This early and large increase, occurring first among children, was anticipated by various models that predicted that the impact of varicella vaccination on HZ incidence would be greatest among cohorts that previously received the most exogenous boosts. Moreover, this post-licensure figure among children with a history of natural (i.e., wild-type) varicella in a study community in which exogenous boosts were rare and asymptomatic endogenous reactions served as the primary boosting mechanism was remarkably similar to the pre-licensure HZ incidence among adults aged 50-59 who demonstrated similar HZ incidence rates in the range of 500 to 550 cases/100,000 p-y. Interestingly, this adult cohort also received the fewest exogenous boosts (relative to younger adults) in the pre-licensure era. Thus, under conditions predominantly influenced by internal boosting, this mechanism may serve to self-limit HZ incidence to 500 cases/100,000 p-y.

Methodology for assessing HZ incidence: stratifying child cohorts

After Goldman resigned in October 2002, three of his papers were peer-reviewed and published in the journal *Vaccine* [13,22,23]. In September 2004, the CDC published a response to the three papers and was critical of the way in which Goldman calculated HZ incidence rates among children [24]. However, properly calculating HZ incidence in a community with moderate varicella vaccination coverage required a different methodology than the approach used in historical studies conducted prior to the implementation of the universal varicella vaccination program. Those studies had simply reported crude incidence rates by combining all children into a single cohort, which was an acceptable approach during the pre-varicella vaccine period. However, after the vaccine was licensed in 1995 and vaccine uptake and coverage rapidly increased in subsequent years, the calculation of a crude (or population) rate was no longer an acceptable method for tracking trends in HZ incidence. Thus, Goldman implemented an approach that stratified children into two separate cohorts: 1) those who received the varicella vaccine and 2) unvaccinated children who had previously contracted wild-type varicella [25]. In this way, the diverse HZ incidence rates could be separately tracked in each of these distinct cohorts. 

By 2000, with approximately 50% of the child population vaccinated, opportunities for unvaccinated children with a history of varicella to gain exogenous boosts to their immunity were greatly diminished. If the Hope-Simpson boosting hypothesis were correct, the HZ incidence rate among unvaccinated children was expected to be (1) much higher than HZ rates in vaccinated children, and (2) greater than HZ rates during the pre-licensure era. Yet, even after the varicella vaccine program matured (from 2000-2002), the CDC advocated the calculation of a single crude HZ incidence rate among all children aged <10 years whether vaccinated or having a history of varicella [24]. This approach yielded a single mean HZ incidence rate of a bimodal distribution that had the effect of concealing the importance of exogenous boosts while masking significantly higher HZ incidence rates--post-varicella vaccine licensure versus pre-licensure--in children with a history of varicella.

Methodology for assessing population HZ incidence rates: capture-recapture

The reported incidence rates in most epidemiological studies (including active surveillance) are extremely poor, missing up to 90% of the cases, with a high degree of variation [26-29]. The unadjusted rates are, at best, lower-bound estimates of the true population rates. Therefore, the options are 1) to report raw varicella and HZ cases, from which population rates are almost uninterpretable, 2) attempt to count every case, which is expensive and slow, or 3) use capture-recapture, which can be a reasonably accurate, quick, and inexpensive approach.

In the VASP, capture-recapture consistently demonstrated 50% reporting completeness (ranging from 43% to 62%) overall child and adolescent age categories and years (2000-2001). In a study published in *JAMA*, CDC/VASP used capture-recapture analysis as evidence that reporting completeness had increased over the study period and, therefore, decreases in varicella incidence were not the result of decreases in the level of reporting [30]. Since the same VASP sites that reported varicella also reported cases of HZ, it was expected that the under-reporting for both would be the same, and capture-recapture analysis confirmed that hypothesis. The US 1990-1994 National Health and Information Survey (NHIS) was used as a criterion standard to investigate if underlying assumptions of capture-recapture application using two ascertainment sources (i.e., schools and medical providers) were reasonable. NHIS age-specific varicella incidence rates compared favorably with those ascertainment-corrected rates reported by VASP and under-reporting of varicella cases among individuals aged 1-19 years was 50% based on the capture-recapture analysis [23].

Additional longitudinal VASP data analyzed and reported by the CDC (2000-2006) corroborated the early cumulative *unadjusted* HZ incidence rates analyzed and reported by Goldman (2000-2003), indicating that the reported *ascertainment-corrected* rates were likely closer estimates of population rates [18]. Both the unadjusted and ascertainment-corrected HZ incidence rates are shown in Table 4 [18-20]. 

By not adjusting for 50% under-reporting of HZ cases to VASP, the HZ incidence rates reported by CDC/VASP were *half the true population rates*. In 2009, when the CDC first published age-specific HZ incidence rates among children and adolescents annually for 2000-2006, CDC/VASP authors compared VASP unadjusted HZ incidence rates to rates reported in other studies using methodology with more exhaustive case collection [19,20], producing potentially misleading comparisons and creating greater variability among HZ incidence rates than would have otherwise been the case [22,23].

Decreased vaccine efficacy after early "honeymoon period"

Exogenous exposures to wild-type varicella appear to have not only boosted cell-mediated immunity to postpone or prevent the reactivation of VZV as HZ in individuals who had previously contracted varicella, but they also augmented efficacy of the varicella vaccine. In 2004, the CDC published a study on the contagiousness of varicella within households, and reported a mean accumulative varicella vaccine efficacy of 78.9% (95% C.I., 69.7% to 85.3%) during 1997-2001 [31]. Unfortunately, the use of a mean vaccine efficacy over several years masked a rapid decline in efficacy as vaccine uptake increased and concealed the importance of exogenous boosts in augmenting the efficacy of the varicella vaccine (Table 5).

**Table 5:** Annual efficacy of single-dose varicella vaccine in households, VASP 1997-2001 stratified by year and CDC-reported mean efficacy Abbreviations: CDC: Centers for Disease Control and Prevention; CI: confidence interval ^a^Efficacy based on household contacts aged <20 years [18]. ^b^Efficacy based on household contacts aged 1-14 years, but neglected transmission resulting from vaccinated (breakthrough) primary cases which increased in proportion from 3.4% in 1997 to 32.9% in 2001 [31]. ^c^37.9% varicella vaccination coverage among children aged 19-35 months ^d^82.1% varicella vaccination coverage among children aged 19-35 months

Year	Vaccine efficacy percentage^a^ (95% CI)	CDC-reported mean efficacy percentage^b^ (95% CI)
1997^c^	87 (75-93)	
1998	94 (83-98)	
1999	96 (83-99)	78.9 (69.7-85.3)
2000^d^	86 (74-92)	
2001	74 (58-84)	

From 1997 to 1999 there was a "honeymoon" effect, where vaccine efficacy increased from 87% to 96%, augmented due to vaccinees receiving exogenous exposures from children infected with wild-type varicella (i.e., contagious children shedding VZV). However, in 2000 and beyond, as more children were vaccinated and the widespread circulation of wild-type varicella declined, exogenous exposures became rare and single-dose vaccine efficacy experienced a >10% annual drop-off in efficacy from 96% in 1999 to 74% in 2001. While not significant at the 95% confidence level (z = 1.96), as noted in the CDC publication [31], the decline was significant at the 94% confidence level (z = 1.88) (Table 5) and further double-digit declines in vaccine efficacy in 2002 and thereafter were statistically significant.

Augmentation of vaccine efficacy by exogenous boosting can only occur when varicella remained endemic, constantly circulating in the environment. Ironically, the "success" of the varicella vaccine in reducing cases of wild-type varicella contributed to the failure of the single-dose vaccine to maintain adequate efficacy to prevent varicella in vaccinated individuals. Thus, starting in 2006, a booster dose of the varicella vaccine was recommended for children aged four to six years. The declining efficacy and necessity for a booster dose dramatically changed the cost-benefit analysis of the universal vaccine program [32]. Based on quantitative results from the annual efficacy study, we would expect that following the booster dose of varicella vaccine administered at ages four to six, IgG levels of anti-VZV specific antibodies would decline each year. Using a conservative figure of 10% annual decline in vaccine efficacy that was observed following the honeymoon effect, we can extrapolate that within 10 years following their last varicella vaccine, substantial numbers of children will no longer be protected against varicella. Affirming the reality of this issue, a recent study among individuals who received two doses of varicella vaccine reported an estimated loss of anti-varicella IgG in 50% of the study group after nine years (95% C.I. 8-9). The authors suggested a third dose of vaccine to restore protection to avoid the risk of future varicella outbreaks [33].

The impacts of universal varicella vaccination in the first decade post-licensure: Other studies

Studies Reporting No Increase in HZ Incidence

Two studies, the Massachusetts Department of Public Health (MDPH) and Group Health Cooperative (GHC) in Seattle, Washington, have been cited multiple times by CDC as having reported no increases in HZ incidence rates in any age group [34-37]. However, these studies had multiple methodological downsides compared to AV-VASP. The MDPH survey consisted of just 4,916 and 3,123 individuals aged 1-19, in years 1999 and 2000 respectively--for a total of 8,039 p-y of observation data. Hence, the small sample size and limited observation time yielded a study design with insufficient statistical power to detect changes in age-specific HZ incidence rates [37]. The other study (GHC) was conducted too early in a population where varicella vaccine uptake had not become sufficiently widespread to impact adult HZ incidence rates [38,39]. Vaccination rates in the Seattle population cohort comprising GHC were lower than the national average. In fact, according to Jumaan et al., "few children (aged 1-9 years) had been vaccinated during 1996 and 1997." CDC authors acknowledged, "The study may have been conducted too early to detect an increase attributed to decrease in exposure to varicella" [38]. The GHC study did, however, report a 67% increase in HZ incidence rates among unvaccinated children aged <10 years-from 87 cases/100,000 p-y in 1996 to 145 cases/100,000 p-y in 2002 [38].

In contrast to the small sample size and limited observation time (i.e., insufficient statistical power) of the MDPH survey [37], AV-VASP's study population included 118,685 individuals aged 1-19 in the years 2000 and 2001 respectively, for a total of 237,370 person-years of observation data [15]. Thus, AV-VASP had an observation time that was nearly 30-fold greater (237,370/8,039) than that of the MDPH study. In contrast to the slow varicella vaccine uptake of the GHC study, AV-VASP had an early startup and rapid uptake in varicella vaccination. By 1999, just four years post-licensure, varicella incidence had already declined by 80% and varicella no longer displayed its characteristic seasonal incidence. Therefore, more meaningful data and conclusions could be drawn from VASP’s HZ surveillance than the MDPH and GHC studies.

Studies Reporting Increased HZ Incidence

In addition to VASP, three studies among adults, two of which were conducted in different U.S. populations using different methods, found statistically significant increases in HZ incidence rates during the first decade post-licensure [15,17,18,40,41]; one study conducted in Australia reported age-standardized HZ incidence had nearly doubled from 2000 to 2012 [40] (Table 6). Annual increases in adult HZ incidence rates ranged from 5.6% to 28.5% dependent on study design and methodology. For children and adolescents, in addition to VASP, three studies conducted in the U.S. and Taiwan also found statistically significant increases in HZ incidence rates during the first decade post-licensure (Table 6) [13,18,38,42]. Annual HZ incidence rate increases ranged from 10.8% to 45.2%, again dependent on various factors such as differences in study methodology and the level of reduction in exogenous exposures within each study population.

**Table 6:** Early post-licensure studies reporting significant percentage HZ incidence rate increases among unvaccinated children, adolescents, and adults ^a^Annual % HZ incidence increase = Overall % HZ incidence rate increase/span; where span = end year – start year ^b^These rates are likely underestimates due to retrospective analysis using HMO databases having methodological limitations. ^c^Varicella vaccination became available in the private sector in Australia in 2000, was publicly funded in Nov. 2005, and varicella vaccine coverage was estimated at 83% among children aged <2 years by March 2011.There was a significant increase in the age-standardized zoster incidence risk among individuals aged <50 years, ranging from 0.58 per 1,000 patients in 1998-1999 to 1.15 per 1,000 patients in 2012 (p<0.05), the change was not uniform. ^d^The overall percentage increase of 226% during a five-year span (1995-2000) is based on the percentage change in the cumulative 1987-1995 true rate of 145 (95% CI 86-228) cases/100,000 p-y reported in the surrogate VASP Adolescent Study and the ascertainment-corrected HZ incidence rate of 472 cases/100,000 p-y reported from active surveillance in 2000 (Table 1). ^e^Overall percentage is based on increase from 87 cases/100,000 p-y in 1996 to 145 cases/100,000 p-y in 2002 among unvaccinated children. ^f^Taiwan implemented a varicella vaccination program in 2004 that required all one-year old children to be vaccinated. ^g^Children in the four years before initiation of mandatory varicella vaccination increased 80% in the two- to three-year follow up post-licensure (IRR=1.8, *P*=0.02 and *P*=0.03, respectively).

Study	Years following varicella vaccine licensure	% HZ incidence rate increase		Location	Study cohort (age in years)
		Overall	Annual^a^		
VASP [15,18]	5 to 6 (2000-2001)	28.5	28.5	Antelope Valley, CA	Adults (20-69)
Yih et al. [17]	4 to 8 (1999-2003)	90	22.5	Massachusetts	Adults (90% >24)
Yawn et al. [41]	1 to 6 (1996-2001)	28.1	5.6^b^	Olmstead County, MN	Adults (>22)
Kelly et al. [40]	0 to 13 (2000-2012)^c^	98	7.5	Victoria, Australia	Adults (<50)
Goldman [13,18]	0 to 5 (1995 & 2000)	226^d^	45.2	Antelope Valley, CA	Children (<10)
Jumaan et al. [38]	1 to 7 (1996-2002)	67^e^	11.2	HMO in WA	Children (<10)
Wen et al. [42]	2 to 3 (2006-2007)^f^	80^g^	26.7	Taiwan	Children (2-7)
Civen et al. [20]	5 to 11 (2000-2006)	65	10.8	Antelope Valley, CA	Adolescents (10-19)

Beyond the First Decade (2005-Present)

In 2008, Reynolds et al., CDC authors, stated there was "evidence from population-based studies that rates of HZ were increasing in the US before the introduction of the varicella vaccine program" [43] and cited longitudinal studies reporting annual HZ incidence rate increases of 2.5% and 3.5% by Donahue et al. [12] and Ragozzino et al. [44], respectively. Regardless of the pre-licensure risk factors responsible for these annual HZ incidence rate increases, they can, for all practical purposes, be ignored since they are very low relative to the higher annual percentage increases reported in the post-licensure period among children, adolescents, and adults with a history of natural varicella.

In 2007, a study by Yawn et al., sponsored by the Mayo Clinic, based on the Rochester Epidemiologic Project (REP) dataset from Homestead County, Minnesota, reported an overall 28.1% increase in the HZ incidence rate (or 5.6% annual increase), ranging from 320 (95% C.I. 290-350) cases/100,000 p-y in 1996/1997 to 410 (95% C.I. 380-440) cases/100,000 p-y in 2000/2001 among adults aged > 22 years [41]. This study concluded, "…vaccination may reduce opportunities for VZV-immune boosting from exposure to natural varicella leading to … increased incidence of HZ among older adults" [41]. However, a more recent analysis by Kawai et al. [45], sponsored by the CDC, using the same REP dataset, contradicted this conclusion and reported HZ incidence rates among all individuals had remained constant over time "after adjusting for age and sex of all ages during a 60-year period ending in 2007."

The Kawai et al. study reported HZ incidence rates among all individuals, including varicella-vaccinated children [45]. With each new birth cohort, vaccinated children had been accumulating in Olmstead County since the varicella vaccine was licensed in 1995. Kawai et al. averaged together both the low HZ incidence rate in that expanding vaccinated cohort and the increasing HZ incidence rate in the unvaccinated adult cohort, thereby creating an artifact--confounded HZ incidence rates--effectively masking the real increase in post-licensure adult HZ incidence rates. 

Since different researchers drew contradictory conclusions from the analysis of the same REP database, this raises the question of publication bias that tends to the favorable outcome: demonstrating that universal varicella vaccination had no impact on the HZ epidemiology. Other CDC-sponsored retrospective studies [46-49] also used outcomes derived from large administrative databases (e.g., MarketScan) to report a constant increase in adult HZ incidence percentage rates and concluded that the universal varicella vaccination program had resulted in no increase in HZ incidence rates over baseline trends. Retrospective studies using data from large administrative databases are not without uncontrolled confounders, significant methodological issues, and controversial results. There is often observable person-time bias that can occur when an individual's onset of HZ (the event of interest) occurs but does not come to the attention of health care [50-51]. Moreover missed person-time, affecting incidence rate calculations, can occur when patients are subject to different secular trends in employment, various economic factors, or the patient seeks a health insurance provider not represented in the MarketScan database. IBM Watson Health, the provider of the MarketScan health databases, describes additional study limitations that include the use of a convenience sample rather than a random population sample. Thus, problems related to monitoring time can occur in the time-period prior to and/or after a patient's onset of HZ due to dynamically changing enrollments, which may impede the study of specific outcomes of HZ incidence.

Predicted Impacts of the Increased Acceleration of HZ Incidence

Depending on the study design and methodology, we have observed post-licensure annual HZ incidence rate increases ranging from 5.6% [41] to 28.5% [15]. The lower percentage increases [41,52] are from retrospective studies using HMO or large administrative databases with many limitations as previously described.

The incidence of HZ in vaccinated adults remains unknown and will need to be studied as the first vaccinated cohort ages. Reactivation of v-VZV has been documented and can lead to HZ [33,53,54]. With vaccine efficacy declining annually and resultant waning of specific anti-VZV immunity, there will continue to be a segment of the population susceptible to breakthrough varicella, who may experience varicella at older ages, when illness is more severe, and who may experience the higher rates of reactivation of wt-VZV as HZ.

## Conclusions

The universal varicella vaccination program in the AV region provided suitable conditions for the CDC-sponsored VASP to gather the first data that could lead to support or rejection of Dr. Hope-Simpson’s 1965 hypothesis that age-specific HZ incidence rates are dependent on the frequency of each cohort’s exposure to individuals shedding wt-VZV. Unfortunately, in study after study, insights on the significance of exogenous boosting were minimized, suppressed, or misrepresented to make unfavorable outcomes of the universal varicella vaccination program appear less concerning than they actually were. This fueled the exogenous boosting debate and prolonged the controversy. Moreover, it created the impression that CDC/VASP manipulated the scientific data to prevent publication of data that could adversely influence immunization rates, regardless of the potential public health consequences.

There is no question that the US universal varicella vaccination program reduced cases of chickenpox. However, any attributed benefits were disproportionately offset by significant HZ incidence rate increases among adults during the first decade post-licensure. Future surveillance and methodologically robust studies will be needed to corroborate the preliminary evidence gathered during the first decade post-licensure in support of Hope-Simpson’s exogenous boosting hypothesis. If exogenous boosts from children shedding wt-VZV are confirmed to be essential to maintaining cell-mediated immunity, universal varicella vaccination may become costly as additional booster doses of the varicella vaccine and/or HZ vaccine are added to provide similar protection that was achieved naturally and at no cost by endemic exogenous exposures in the pre-licensure era.

## References

[1] Hope-Simpson RE (1965). The nature of herpes zoster: a long-term study and a new hypothesis. Proc R Soc Med.

[2] Arvin AM, Koropchak CM, Wittek AE (1983). Immunologic evidence of reinfection with varicella-zoster virus. J Infect Dis.

[3] Brisson M, Gay NJ, Edmunds W J, Andrews NJ (2002). Exposure to varicella boosts immunity to herpes-zoster: implications for mass vaccination against chickenpox. Vaccine.

[4] Gershon AA, LaRussa P, Steinberg S, Mervish N, Lo SH, Meier P (1996). The protective effect of immunologic boosting against zoster: an analysis in leukemic children who were vaccinated against chickenpox. J Infect Dis.

[5] Salleras M, Domínguez A, Soldevila N (2011). Contacts with children and young people and adult risk of suffering herpes zoster. Vaccine.

[6] Solomon BA, Kaporis AG, Glass AT, Simon SI, Baldwin HE (1998). Lasting immunity to varicella in doctors study (L.I.V.I.D. study). J Am Acad Dermatol.

[7] Terada K, Hiraga Y, Kawano S, Kataoka N (1995). Incidence of herpes zoster in pediatricians and history of reexposure to varicella-zoster virus in patients with herpes zoster [article in Japanese]. Kansenshogaku Zasshi.

[8] Thomas SL, Wheeler JG, Hall AJ (2002). Contacts with varicella or with children and protection against herpes zoster in adults: a case-control study. Lancet.

[9] Food and Drug Administration (2021). Food and Drug Administration. Summary for basis of approval: Merck & Co., Varicella Virus Vaccine Live, VARIVAX®. 1995.

[10] Krause PR, Klinman DM (1995). Efficacy, immunogenicity, safety, and use of live attenuated chickenpox vaccine. J Pediatr.

[11] Lieu TA, Cochi SL, Black SB (1994). Cost-effectiveness of a routine varicella vaccination program for US children. JAMA.

[12] Donahue JG, Choo PW, Manson JE, Platt R (1995). The incidence of herpes zoster. Arch Intern Med.

[13] Goldman GS (2003). Varicella susceptibility and incidence of herpes zoster among children and adolescents in a community under active surveillance. Vaccine.

[14] Goldman GS, King PG (2013). Review of the United States universal varicella vaccination program: Herpes zoster incidence rates, cost-effectiveness, and vaccine efficacy based primarily on the Antelope Valley Varicella Active Surveillance Project data. Vaccine.

[15] Maupin T, Goldman GS, Peterson C, Mascola L (2002). Annual summary, Antelope Valley Varicella Active Surveillance Project (VASP), Los Angeles County Department of Health Services (LADHS); Centers for Disease Control and Prevention (CDC). Cooperative Agreement No. U66/CCU911165-10.

[16] Tilling K (2001). Capture-recapture methods--useful or misleading?. Int J Epidemiol.

[17] Yih WK, Brooks DR, Lett SM, Jumaan AO, Zhang Z, Clements KM, Seward JF (2005). The incidence of varicella and herpes zoster in Massachusetts as measured by the Behavioral Risk Factor Surveillance System (BRFSS) during a period of increasing varicella vaccine coverage, 1998-2003. BMC Public Health.

[18] Goldman GS (2005). Universal varicella vaccination: efficacy trends and effect on herpes zoster. Int J Toxicol.

[19] Civen R, Marin M, Zhang J, Abraham A, Harpaz R, Mascola L, Bialek SR (2016). Update on incidence of herpes zoster among children and adolescents after implementation of varicella vaccination, Antelope Valley, CA, 2000 to 2010. Pediatr Infect Dis J.

[20] Civen R, Chaves SS, Jumaan A, Wu H, Mascola L, Gargiullo P, Seward JF (2009). The incidence and clinical characteristics of herpes zoster among children and adolescents after implementation of varicella vaccination. Pediatr Infect Dis J.

[21] Tseng HF, Smith N, Marcy SM, Sy LS, Jacobsen SJ (2009). Incidence of herpes zoster among children vaccinated with varicella vaccine in a prepaid health care plan in the United States, 2002-2008. Pediatr Infect Dis J.

[22] Goldman GS (2003). Incidence of herpes zoster among children and adolescents in a community with moderate varicella vaccination coverage. Vaccine.

[23] Goldman GS (2003). Using capture-recapture methods to assess varicella incidence in a community under active surveillance. Vaccine.

[24] Jumaan A, Schmid DS, Gargiullo P, Seward J (2004). Scientific commentary. Vaccine.

[25] Goldman GS (2004). Response to Letter to the Editor by Jumaan: Goldman's role in the Varicella Active Surveillance Project. Vaccine.

[26] Deming WE (1991[1986]). Out of the Crisis. https://www.worldcat.org/title/out-of-the-crisis/oclc/926776548.

[27] Hook EB, Regal RR (1992). The value of capture-recapture methods even for apparent exhaustive surveys. The need for adjustment for source of ascertainment intersection in attempted complete prevalence studies. Am J Epidemiol.

[28] McCarty DJ, Tull ES, Moy CS, Kwoh CK, LaPorte RE (1993). Ascertainment corrected rates: applications of capture-recapture methods. Int J Epidemiol.

[29] Thacker SB, Berkelman RL (1988). Public health surveillance in the United States. Epidemiol Rev.

[30] Seward JF, Watson BM, Peterson CL (2002). Varicella disease after introduction of varicella vaccine in the United States, 1995-2000. JAMA.

[31] Seward JF, Zhang JX, Maupin TJ, Mascola L, Jumaan AO (2004). Contagiousness of varicella in vaccinated cases: a household contact study. JAMA.

[32] Zhou F, Santoli J, Messonnier ML (2005). Economic evaluation of the 7-vaccine routine childhood immunization schedule in the United States, 2001. Arch Pediatr Adolesc Med.

[33] Bianchi FP, Tafuri S, Larocca AM, Germinario CA, Stefanizzi P (2021). Long -term persistence of antibodies against varicella in fully immunized healthcare workers: an Italian retrospective cohort study. BMC Infect Dis.

[34] Edmunds J, Brisson M (2002). Potential changes in zoster epidemiology with childhood immunization. 42nd Interscience Conference on Antimicrobial Agents and Chemotherappy (ICAAC).

[35] Roche P, Blumer C, Spencer J (2002). Surveillance of viral pathogens in Australia--varicella-zoster virus. Commun Dis Intell Q Rep.

[36] Seward JF, Jumaan AO, Galil K, Wharton M (2002). Varicella vaccine and shingles—reply. JAMA.

[37] Yih WK, Brooks DR, Clements KM, Lett SM, Jumaan A, Seward J (2003). The Incidence of varicella and herpes zoster in Massachusetts as measured by the Behavioral Risk Factors Surveillance System (BRFSS) during a period of increasing varicella vaccine coverage, 1998-2000. The 37th National Immunization Conference of CDC, March 19.

[38] Jumaan AO, Yu O, Jackson LA, Bohlke K, Galil K, Seward JF (2005). Incidence of herpes zoster, before and after varicella-vaccination-associated decreases in the incidence of varicella, 1992-2002. J Infect Dis.

[39] Whitley RJ (2005). Changing dynamics of varicella-zoster virus infections in the 21st century: the impact of vaccination. J Infect Dis.

[40] Kelly HA, Grant KA, Gidding H, Carville KS (2014). Decreased varicella and increased herpes zoster incidence at a sentinel medical deputising service in a setting of increasing varicella vaccine coverage in Victoria, Australia, 1998 to 2012. Euro Surveill.

[41] Yawn BP, Saddier P, Wollan PC, St Sauver JL, Kurland MJ, Sy LS (2007). A population-based study of the incidence and complication rates of herpes zoster before zoster vaccine introduction. Mayo Clin Proc.

[42] Wen SY, Liu WL (2015). Epidemiology of pediatric herpes zoster after varicella infection: a population-based study. Pediatrics.

[43] Reynolds MA, Chaves SS, Harpaz R, Lopez AS, Seward JF (2008). The impact of the varicella vaccination program on herpes zoster epidemiology in the United States: a review. J Infect Dis.

[44] Ragozzino MW, Melton LJ 3rd, Kurland LT, Chu CP, Perry HO (1982). Population-based study of herpes zoster and its sequelae. Medicine (Baltimore).

[45] Kawai K, Yawn BP, Wollan P, Harpaz R (2016). Increasing incidence of herpes zoster over a 60-year period from a population-based study. Clin Infect Dis.

[46] Harpaz R (2019). Do varicella vaccination programs change the epidemiology of herpes zoster? A comprehensive review, with focus on the United States. Expert Rev Vaccines.

[47] Harpaz R, Leung JW (2019). The epidemiology of herpes zoster in the United States during the era of varicella and herpes zoster vaccines: changing patterns among children. Clin Infect Dis.

[48] Harpaz R, Leung JW (2019). The epidemiology of herpes zoster in the United States during the era of varicella and herpes zoster vaccines: changing patterns among older adults. Clin Infect Dis.

[49] Harpaz R, van Hoek AJ (2018). Point-counterpoint: the Hope-Simpson hypothesis and its implications regarding an effect of routine varicella vaccination on herpes zoster incidence. J Infect Dis.

[50] Prada-Ramallal G, Takkouche B, Figueiras A (2019). Bias in pharmacoepidemiologic studies using secondary health care databases: a scoping review. BMC Med Res Methodol.

[51] Kolossváry E, Ferenci T, Kováts T (2020). Potentials, challenges, and limitations of the analysis of administrative data on vascular limb amputations in health care. Vasa.

[52] Wolfson LJ, Daniels VJ, Altland A, Black W, Huang W, Ou W (2020). The impact of varicella vaccination on the incidence of varicella and herpes zoster in the United States: updated evidence from observational databases, 1991-2016. Clin Infect Dis.

[53] Chaves SS, Haber P, Walton K, Wise RP, Izurieta HS, Schmid DS, Seward JF (2008). Safety of varicella vaccine after licensure in the United States: experience from reports to the vaccine adverse event reporting system, 1995-2005. J Infect Dis.

[54] Galea SA, Sweet A, Beninger P, Steinberg SP, Larussa PS, Gershon AA, Sharrar RG (2008). The safety profile of varicella vaccine: a 10-year review. J Infect Dis.

