# Correction: Insights on the Impact of External and Internal Boosting on Varicella-Zoster Virus Reactivation Based on Evidence From the First Decade of the United States Universal Varicella Vaccination Program

**DOI:** 10.7759/cureus.c47

**Published:** 2021-09-07

**Authors:** Gary S Goldman

**Affiliations:** 1 Research, Independent Computer Scientist, Bogue Chitto, USA

This article was published with an incorrect reference: #36 was originally submitted as 'Seward JF, Jumaan AO, Galil K, Wharton M: Varicella vaccine and shingles—reply. JAMA. 2002, 287:2211-2212.' but due to the inclusion of an incorrect reference, it was automatically (and incorrectly) changed to the following: 'Stevenson LW: Treatment of congestive heart failure. JAMA. 2002, 287:2209-10.'

The incorrect reference #36 (Stevenson) has been replaced with the original, correct reference (Seward et al). Cureus deeply regrets the error and any confusion caused as a result.

 

